# EEG Correlates of Attentional Load during Multiple Object Tracking

**DOI:** 10.1371/journal.pone.0022660

**Published:** 2011-07-26

**Authors:** Heather Sternshein, Yigal Agam, Robert Sekuler

**Affiliations:** 1 Department of Neurobiology, Harvard Medical School, Boston, Massachusetts, United States of America; 2 Athinoula A. Martinos Center for Biomedical Imaging, Harvard Medical School, Boston, Massachusetts, United States of America; 3 Volen Center for Complex Systems, Brandeis University, Waltham, Massachusetts, United States of America; Central Queensland University, Australia

## Abstract

While human subjects tracked a subset of ten identical, randomly-moving objects, event-related potentials (ERPs) were evoked at parieto-occipital sites by task-irrelevant flashes that were superimposed on either tracked (Target) or non-tracked (Distractor) objects. With ERPs as markers of attention, we investigated how allocation of attention varied with tracking load, that is, with the number of objects that were tracked. Flashes on Target discs elicited stronger ERPs than did flashes on Distractor discs; ERP amplitude (0–250 ms) decreased monotonically as load increased from two to three to four (of ten) discs. Amplitude decreased more rapidly for Target discs than Distractor discs. As a result, with increasing tracking loads, the difference between ERPs to Targets and Distractors diminished. This change in ERP amplitudes with load accords well with behavioral performance, suggesting that successful tracking depends upon the relationship between the neural signals associated with attended and non-attended objects.

## Introduction

Multiple Object Tracking (MOT) is used widely in studies of visual attention's dynamics and flexibility [1], [2]. In MOT, a subset of identical, stationary objects, discs for example, is temporarily marked with a visual tag, such as a distinctive color. This distinguishing tag is then withdrawn, and all the identical objects in the entire set, ones that had been marked as well as ones that had not been marked, move about randomly and independently of one another [3]. For the several seconds during which all objects are moving, subjects try to keep track of those objects that originally had been tagged. The tracked objects are called Targets; the non-tracked objects are called Distractors. As one might imagine from this brief description, the task can be quite challenging, particularly when objects move rapidly or are numerous [4], [5]. The task's dynamic nature demands that attention be maintained over the entire time that objects are in motion [2], although there seems to be no consensus about the attentional mechanisms that are recruited by the task [2], [3], [6].

Functional magnetic resonance imaging (fMRI) has identified regions of the brain that may support performance of MOT. These regions include the inferior parietal lobule, the intraparietal sulcus, and frontal areas [7], [8]. Such regions show increased activation as the number of objects that must be tracked is increased. This load-dependent increase in activation has been interpreted as a correlate of increased attentional demands. However, its limited temporal resolution prevents fMRI from directly connecting variation in activation to the way that attention is allocated among individual moving objects. Recent theoretical formulations of the MOT task [2] suggest that establishing such a connection is a critical prerequisite for understanding the mechanisms that support MOT.

Therefore we turned to electroencephalography (EEG) as a way to examine the assignment of attention to individual objects, Targets and Distractors. We reasoned that EEG's good temporal resolution would allow us to isolate physiological correlates of attention to individual Targets and Distractors, which would be an important step toward understanding subjects' successes and failures in performing MOT. More specifically, within a signal detection framework [9], [10], we hypothesize that distinguishing Targets from Distractors depends upon the differential attention devoted to objects in each class. This hypothesis implies that when conditions reduce that differential in attention, the task grows more difficult, and subjects make more errors, mistaking Distractors for Targets.

Recently, ERPs collected from subjects who were performing the MOT task led Drew *et al.* [11] to argue that attention to multiple moving objects involves an enhancement of attended disc locations rather than a suppression of attention to non-target, distractor locations. It is important to note that in that study, each object's moved quite slowly, at ∼1°/second. In contrast, most other studies of MOT have used considerably higher object speeds, ranging from three to more than 20 times that value [1]–[3], [12]–[15], and MOT performance depends strongly upon the speed with which tracked objects move. More importantly, speeds as low as 1°/second may be relatively ineffective in activating motion mechanisms [16]. For example, velocity thresholds, an index of sensitivity to motion, are low (about 6%) and constant over the range of velocities [17], [18] that are most commonly used in studies of MOT. In contrast, that same index of motion sensitivity declines dramatically for velocities slower than or faster than that range, *e.g.*, 1°/second. Further, Drew et al. [11] did not investigate the effect of variation in attentional load, that is, the number of objects comprising the target subset. Throughout their experiment, subjects had to track only two objects in a set of four total moving objects. The combination of so few target objects and the slowness of their motion opens the possibility that subjects simply switched attention back and forth between the objects [2], rather than tracking multiple objects at once. Therefore, it is hard to draw firm comparisons between these results and the ones obtained earlier, with fMRI.

## Results and Discussion

**Figure 1:**
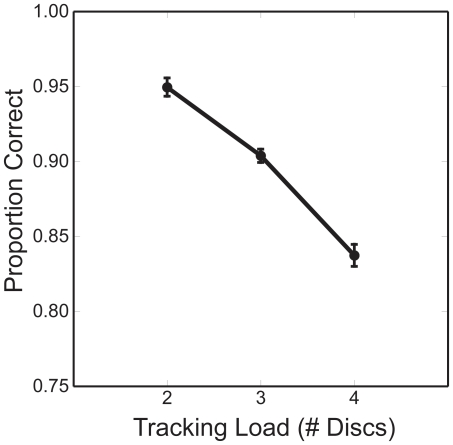
Behavioral performance as a function of the number of discs being tracked. Error bars show ±1 within-subject standard errors.

Behavioral performance was defined by the proportion of perfect trials, that is, trials on which every disc that had been marked at trial's start was correctly selected by the subject after motion had ceased. The proportion of perfect trials decreased monotonically with increasing load (one-way ANOVA (*F*(2,38) = 57.77,p<0.001; Figure 1).

Each subject performed a fixation-check task at or above 90%, suggesting good compliance with the instructions to maintain fixation. The mean and standard deviation of the proportion correct were 0.92 and 0.056, respectively. Fixation-check trials were excluded from the analysis of ERPs.

**Figure 2:**
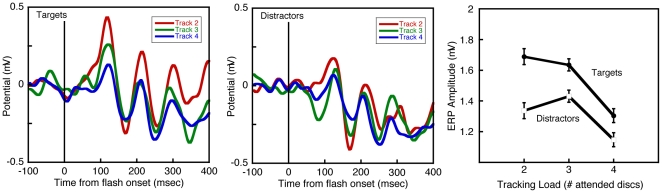
Left and Center Panels: Grand-average event-related potentials elicited by task-irrelevant flashes on a Target disc (Left) or on a Distractor disc (Center). In each panel, ERPs obtained while subjects tracked 2 discs are shown in red; ERPs obtained while subjects tracked 3 discs are shown in green; and ERPs obtained while 4 discs were being tracked are shown in blue. The vertical line in each panel marks the onset of the flash. Right Panel: log ERP amplitude (maximum–minimum) for tracking loads of 2, 3 and 4 discs. ERPs to flashes on Target discs are represented by black circles; ERPs evoked by flashes on Distractor discs are shown with white squares. Error bars represent within-subject SEM.

Figure 2 shows grand average ERPs for the three tracking loads. Each ERP was time-locked to the onset of the task-irrelevant flash. A bootstrap analysis was applied to these ERP amplitudes. ERP amplitude declined with load for both Targets (p<0.0001) and Distractors (p<0.02). However, a second bootstrap analysis showed that load had a different effect for Targets than for Distractors (p<0.02).

The relative magnitudes of ERPs to the two kinds of stimuli suggest that Targets receive more attention than Distractors do, and that the amount of attention allocated to each tracked disc falls with increasing tracking load. Most important for understanding MOT performance, the *difference* between ERPs evoked by flashed Target and flashed Distractor discs narrows as additional items must be tracked. This decrease in ERP amplitudes (and narrowing between ERPs of target and distractor discs) with load accords well with decreased behavioral performance with increased load, suggesting that successful tracking depends upon the relationship between the neural signals associated with attended and non-attended objects. This shrinking difference between ERPs to the two kinds of stimuli could explain the systematic increase in errors with tracking load (Figure 1). It is noteworthy that the response to flashed Targets in one condition can fall below the response to flashed Distractors in another, for example, flashed Targets in the highest load condition elicited smaller responses than flashed Distractors in the lowest load condition. It seems unlikely, then, that reduced attention to Targets alone can explain the decline in MOT performance with load. Rather, as tracking load grows, it becomes more difficult for subjects to apportion attentional resources in a way that preserves a sufficient advantage for Targets over Distractors [19]. As with other cognitive tasks in which signal must be discriminated from noise [9], MOT requires the use of probabilistic evidence to partition stimuli into a few categories, here, the categories of Targets and Distractors. This overall framework could help to explain why errors increased in parallel with the shrinking difference between the ERP amplitudes for the two types of stimuli. Specifically, as the two mean values of allocated attention (relative to their standard deviations) approached each other, non-tracked objects would be increasingly mistaken for tracked ones.

The results presented here replicate the difference in ERP target and distractor amplitude when tracking two discs reported by Drew *et al.* [11] but with disc speeds more comparable to other MOT studies. Further, our results describe how the ERP difference changes with attentional load, that is, the number of objects comprising the target subset. Although many studies [20]–[23] have shown that attended locations and objects elicit larger ERPs than unattended ones, only one other study has examined the effects of attention to multiple *moving* objects [24]. Although we did not examine individual ERP components, our measure of neural activity included both the P1 and N1 component, which are enhanced with attention to locations and objects.

We cannot rule out the possibility that the difference between the lowest and highest load conditions arose from a change in attentional strategy between conditions. In the track 2 condition, subjects may have simply switched attention back and forth between two objects [2], rather than tracking multiple objects at once. Indeed, Doran and Hoffman [24] recently suggested that ERP amplitudes change with variations to the MOT task (and subsequent difficulty) that result from shifting attentional strategies. Incorporating eye tracking measures in future studies could reveal whether subjects are indeed changing strategies across loads.

We should acknowledge that even on trials with 100% accuracy in identification of Targets, the opportunity for some correct guesses makes it impossible to guarantee that only Targets had actually been tracked. In fact, there are questions about the accuracy of any single behavioral method that is used to assess the number of tracked objects on any trial [25]. That caveat applies to the “mark-all” method used in our study, as well as to the common, “probe-one” method in which subjects indicate whether some particular item belongs to the Target set. These methods do, however, produce values that are at least proportional to the number of items actually tracked, which gives some confidence in the load-induced decline we found in tracking performance. The imprecision in defining how many Targets were tracked means that on some number of nominally “perfect” trials a flash on a Target disc would have been delivered to a disc that was actually not tracked, and a flash delivered on a Distractor disc would have been delivered to a disc that was tracked. This limitation on the equation between “Target” and “tracked”, means that the differences in ERPs that we saw for Targets and Distractors may define a lower bound on the difference in which we were interested.

Finally, our results demonstrate the value of using ERP as an index of the differential attention allocated to items in a Target set and to items in a Distractor set. In fact, the approach described here can be usefully extended in order to study the basis of practice-dependent improvement in MOT [26], and the deployment of attentional resources by members of populations whose MOT performance is atypical, *e.g.*, older adults [14], patients with schizophrenia [27], and habitual video-game players [28].

## Materials and Methods

### Subjects

Twenty-two right-handed subjects (14 female; age range: 19–30) with normal or corrected to normal vision participated. All subjects were recruited from the student population of Brandeis University, and gave written informed consent to a protocol approved by Brandeis University's Committee for the Protection of Human Subjects. Subjects were compensated monetarily for their time. Each subject participated in a pair of 2-hour sessions, with 300 experimental trials in each session. All subjects provided written informed consent in accordance with the Declaration of Helsinki, and were compensated. The protocol was approved by the Brandeis University Committee for the Protection of Human Subjects.

### Stimuli and Procedure

**Figure 3:**
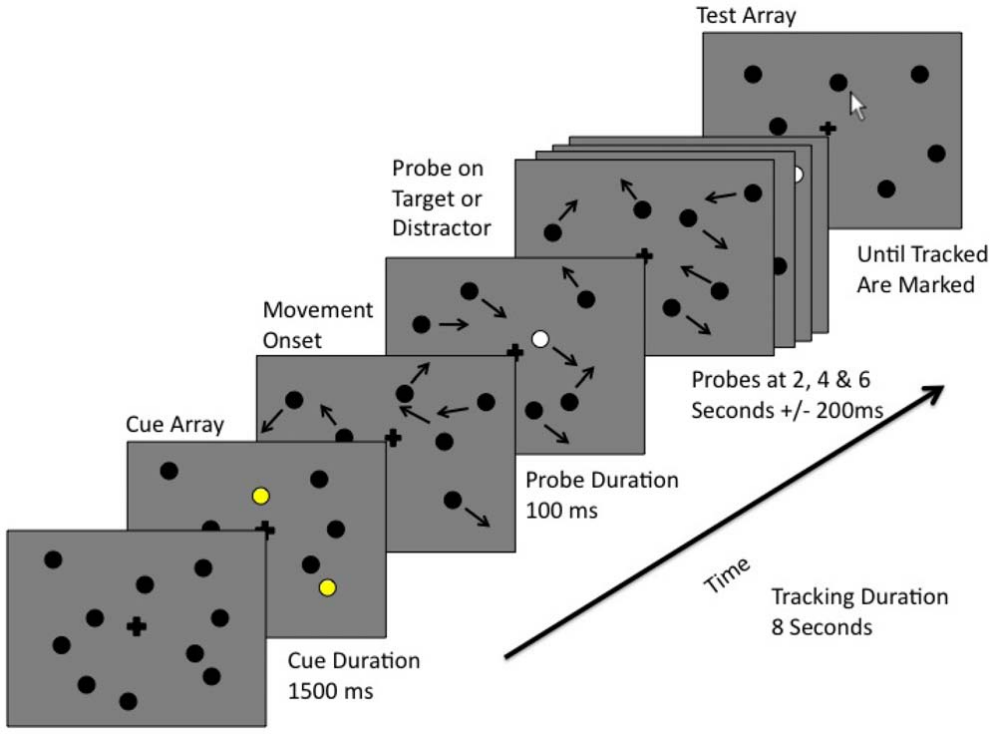
The sequence of events comprising an MOT trial. At the start of each trial, 10 black discs (each 1° in diameter) appeared on a uniform gray background along with a central fixation cross. All 10 discs were presented within a 15°×15° notional bounding box that was centered on the computer display. Then, a randomly-chosen subset (either two, three, or four) of the 10 discs temporarily changed color, from black to yellow and back to black. This brief color change designated the discs that the subject would subsequently have to track. After all the discs had been restored to their original, black color, they started to move, each on an independent random trajectory. If two discs came within a radius of each other, both discs reversed direction. If a disc's motion would bring it to the defined edge of the tracking field, the disc's direction changed, with the disc's angle of reflection set equal to what would have been the angle with which it would have hit the edge. To elicit event related potentials (ERPs), task-irrelevant flashes were briefly (100 ms) superimposed on one of the discs, at 2, 4, and 6 seconds ±200 ms. Flashes were randomly distributed between Target and Distractor discs, with the two types being flashed equally often. After an 8 second tracking period, all discs stopped moving. Subjects then used a computer mouse to select the target discs, that is, the discs that were supposed to have been tracked.

Subjects viewed the display binocularly from a distance of ∼57 cm. The stimuli that they saw were generated and displayed using Matlab 7 and Psychtoolbox extensions on a CRT monitor [29]. The Multiple Object Tracking task used here was generally as described in a previous publication from our laboratory [14] describing age-related changes in MOT performance. Figure 3 depicts the sequence of events that comprised an MOT trial. On each trial, 10 black discs (1° visual angle/disc, 1 cd/m^2^) were presented within a 15°\15° box centered on a gray background (41 cd/m^2^). The initial locations of the discs were chosen randomly, except that no disc was permitted to lie within 1° visual angle of any other disc. Then, after 750 ms, a subset of these discs alternated between black and yellow (195 cd/m^2^) at 4 Hz for 1.5 seconds. This marked the color-alternating discs as the ones that were to be tracked. The marked subset consisted of two, three, or four discs, which have been shown to correspond to low, medium, and high attentional load trials, respectively [30]. When all discs had returned to their original color (black), they then started moving in randomly-chosen directions (at 10°/second). A portion of each disc's trajectory was predictable in that a disc did not change direction unless it encountered another disc or the edge of the display area. If a disc came within 0.5° visual angle of another disc or the display area's boundary, the disc abruptly reversed direction. During the first second of the 8-second tracking period, objects accelerated gradually from 0 to 10°/s. This slow acceleration eliminated the possibility that an abrupt onset of motion would cause attentive tracking to fail before the presentation of the first flash.

Of each session's 300 trials, subjects tracked two objects on 90 trials, three objects on 90 trials, and four objects on 120 trials. The additional trials on which four objects were tracked were intended to compensate for the expected increase in errors in that condition. The number of tracked objects was randomized over trials. A single white task-irrelevant flash (100 ms duration, 224 cd/m^2^) was presented, either on a Target or Distractor disc, at 2, 4, and 6 seconds ±200 ms into the eight-second long tracking portion of each trial. The random jitter in timing was introduced to dissuade subjects from anticipating each flash. Flashes were randomized, such that on any trial, flashes occurred on any possible combination of target and distractor discs.

Across the entire experiment for each load, 50% of task-irrelevant flashes were superimposed on target discs, and 50% were superimposed on distractor discs. Subjects were informed that flashes were not relevant, and that the flashes would neither help nor hinder their tracking performance. When the motion of the discs ceased, the subject used the mouse cursor to select each disc that he or she believed had been marked at the trial's start, by the color alternation. Before their first session, subjects completed 21 practice trials to become familiar with the task.

When a subject selected and clicked on a disc, that disc disappeared from view. This eased subjects' task by eliminating the need to keep track of which discs had already been selected. The next trial began after the subject had clicked on as many discs as had been marked at the start of a trial (two, three, or four discs) and indicated, with a button press, readiness to proceed. The interval between trials was controlled by the subject.

Our analysis of ERPs required the availability of many trials representing comparable levels of behavioral performance. For this purpose, it was convenient to restrict the ERP analysis to trials on which every identification was correct. The choice to use only “perfect” trials was made partly because after subjects lose track of some Target disc, they might mistakenly track a Distractor disc in its place. If this happened, the distinction between types of trials –Target flashed vs. Distractor flashed– would have been undermined. To generate enough “perfect” trials for the ERP analysis, we devised displays whose characteristics, particularly the gradual onset of motion and the rules forbidding overlap of the discs, would produce relatively good behavioral performance, even with the highest number of Targets that we tested. It should be noted, that even with good behavioral performance, all of our subjects reported that tracking 4 discs as compared to 2 discs was much harder.

To verify that subjects maintained fixation, on 10% of the trials they performed a challenging orientation-discrimination task. These fixation-check trials, which occurred randomly, began as ordinary MOT trials, which rendered them unpredictable. This unpredictability was meant to promote compliance with fixation instructions on all trials. On fixation-check trials, subjects saw a short (45′) dark gray line that was briefly flashed at fixation. The brief (200 msec) presentation of this line occurred at random times within the fixation-check trial. From trial to trial, the line′s orientation varied randomly between vertical and an orientation 10° clockwise from vertical. The subject pushed one of two buttons to signal whether the line was vertical or rotated from vertical.

### EEG Recording and Analysis

We recorded electroencephalographic signals using a high-density, 128-electrode array (Electrical Geodesics, Eugence, OR). The electrode array included bipolar channels located above and below each eye, and one bipolar eye channel located near the outer canthus of each eye. Sensor signals were sampled at 250 Hz, and recorded for offline analysis. BESA (MEGIS Software GmbH, Munich) was used to process and re-reference data to the grand average. Follow-on analysis was done in Matlab (Mathworks, Natick, MA). For all analyses, data were first notch-filtered at 60 Hz, and then bandpass filtered between 0.1 and 30 Hz. Blink artifacts were eliminated by rejecting epochs in which the difference between the maximum and minimum voltage at any channel, including the ocular channels, during an entire ERP epoch (100 ms pre- and 250 ms post-flash onset) exceeded 120 μV. Epochs containing such artifacts were excluded from analysis.

ERPs to the task-irrelevant 100 ms flashes were measured by averaging signals across sensors positioned over occipital and parietal areas. The sites, as defined in the 10/20 system, were O1, Oz, and O2, and P3, Pz, P4, P7, and P8 (for a similar approach see [31]–[34]). We expected these areas to be most activated by the task-irrelevant flashes (eliciting visually evoked event-related potentials) and to be activated during MOT. Data were averaged across trials, and then each subject's mean ERP for each condition was computed and analyzed. ERPs were baseline-corrected by subtracting from each the mean potential during the 100 msec prior to the onset of the flash.

Our dependent measure was ERP amplitude, i.e., the difference between the maximum and minimum potentials during the period of 0 to 250 ms relative to flash onset. This epoch was chosen because it contains components that are strongly influenced by both spatial and object attention [20]–[23] Because our amplitude measure is inherently non-normal, we used nonparametric statistical tests to assess the reliability of the amplitude differences across load conditions (for other examples of the use of nonparametric statistics with ERP data, see [34]–[36]). We carried out two independent bootstrap procedures: one to evaluate the effect of load for Target and Distractor discs, and another to evaluate how load-dependent amplitude changes were affected by the type of the disc on which the flash occurred (Target or Distractor). To examine the load effect, we created 10,000 random data sets reflecting the null hypothesis that the slope of the amplitude regressed against load would be zero. For each simulated dataset, load was randomly assigned (with replacement) to each subject's individual ERPs. In each such dataset, the amplitudes of subjects' randomized ERPs were regressed against load, yielding a distribution of 10,000 regression slope values. The position, within the bootstrap distribution, of the slope derived from regression of the actual ERP amplitude against load was used to determine the probability that such a slope would occur by chance. To test whether the load effect differed between Targets and Distractors, we calculated the load-based regression slope for each subject and disc type. These slopes were then randomly assigned to disc types, namely “Target” or “Distractor”, reflecting the null hypothesis that the effect of load is not different between targets and distractors. The difference between the slopes was tested using the same bootstrap procedure as with the load effect, this time the independent variable being disc type. All P values reported below for nonparametric tests reflect the number of simulations out of 10,000 that exceeded the slope of the actual data (divided by two to adjust for a two-tailed test).

## References

[1] Cavanagh P, Alvarez GA (2005). Tracking multiple targets with multifocal attention.. Trends in Cognitive Science.

[2] Oksama L, Hyönä J (2008). Dynamic binding of identity and location information: a serial model of multiple identity tracking.. Cognitive Psychology.

[3] Pylyshyn ZW, Storm RW (1988). Tracking multiple independent targets: evidence for a parallel tracking mechanism.. Spatial Vision.

[4] Shim WM, Alvarez GA, Jiang YV (2008). Spatial separation between targets constrains maintenance of attention on multiple objects.. Psychonomic Bulletin & Review.

[5] Franconeri SL, Lin JY, Pylyshyn ZW, Fisher B, Enns JT (2008). Evidence against a speed limit in multiple-object tracking.. Psychonomic Bulletin & Review.

[6] Kazanovich Y, Borisyuk R (2006). An oscillatory neural model of multiple object tracking.. Neural Computing.

[7] Culham JC, Brandt SA, Cavanagh P, Kanwisher NG, Dale AM (1998). Cortical fMRI activation produced by attentive tracking of moving targets.. Journal of Neurophysiology.

[8] Jovicich J, Peters RJ, Koch C, Braun J, Chang L (2001). Brain areas specific for attentional load in a motion-tracking task.. Journal of Cognitive Neuroscience.

[9] Swets JA (1996). Signal Detection Theory and ROC Analysis in Psychology and Diagnostics..

[10] Wickens TD (2001). Elementary signal detection theory..

[11] Drew T, McCollough AW, Horowitz TS, Vogel EK (2009). Attentional enhancement during multipleobject tracking.. Psychonomic Bulletin & Review.

[12] Scholl BJ, Pylyshyn ZW, Feldman J (2001). What is a visual object? evidence from target merging in multiple object tracking.. Cognition.

[13] Trick LM, Perl T, Sethi N (2005). Age-related differences in multiple-object tracking.. Journal of Gerontology, B: Psychological Science, Social Science.

[14] Sekuler R, McLaughlin C, Yotsumoto Y (2008). Age-related changes in attentional tracking of multiple moving objects.. Perception.

[15] Howe PDL, Horowitz TS, Morocz IA, Wolfe JM, Livingstone MS (2009). Using fMRI to distinguish components of the multiple object tracking task.. Journal of Vision.

[16] McKee SP, Watamaniuk SNJ (1994). Visual Detection of Motion..

[17] De Bruyn B, Orban GA (1988). Human velocity and direction discrimination measured with random dot patterns.. Vision Research.

[18] Orban GA, de Wolf J, Maes H (1984). Factors inuencing velocity coding in the human visual system.. Vision Research.

[19] Ma WJ, Huang W (2009). No capacity limit in attentional tracking: evidence for probabilistic inference under a resource constraint.. Journal of Vision.

[20] Luck SJ (1995). Multiple mechanisms of visual-spatial attention: recent evidence from human electrophysiology.. Behav Brain Res.

[21] Di Russo F, Martínez A, Hillyard SA (2003). Source analysis of event-related cortical activity during visuo-spatial attention.. Cereb Cortex.

[22] Hopfinger J, Luck S, Hillyard A (2004). The Cognitive Neurosciences Cambridge.

[23] Martínez A, Teder-Sälejärvi W, Vazquez M, Molholm S, Foxe JJ (2006). Objects are highlighted by spatial attention.. Journal of Cognitive Neuroscience.

[24] Doran MM, Hoffman JE (2010). The role of visual attention in multiple object tracking: evidence from erps.. Atten Percept Psychophys.

[25] Hulleman J (2005). The mathematics of multiple object tracking: from proportions correct to number of objects tracked.. Vision Research.

[26] Makovski T, Vázquez GA, Jiang YV (2008). Visual learning in multiple-object tracking.. PLoS ONE.

[27] Kelemen O, Nagy O, Mátyássy A, Bitter I, Benedek G (2007). How well do patients with schizophrenia track multiple moving targets?. Neuropsychology.

[28] Green CS, Bavelier D (2006). Enumeration versus multiple object tracking: the case of action video game players.. Cognition.

[29] Brainard DH (1997). The psychophysics toolbox.. Spatial Vision.

[30] Zelinsky GJ, Todor A (2010). The role of "rescue" saccades in tracking objects through occlusions.. Journal of Vision.

[31] Vogel EK, Machizawa MG (2004). Neural activity predicts individual differences in visual working memory capacity.. Nature.

[32] Vogel EK, McCollough AW, Machizawa MG (2005). Neural measures reveal individual differences in controlling access to working memory.. Nature.

[33] Zhang W, Luck SJ (2009). Sudden death and gradual decay in visual working memory.. Psychological Science.

[34] Agam Y, Huang J, Sekuler R (2010). Neural correlates of sequence encoding in visuomotor learning.. Journal of Neurophysiology.

[35] Price GW (2000). Interactive ERP recording increases the amplitude of the endogenous P300 peak in schizophrenia.. Schizophrenia Research.

[36] Stroganova TA, Orekhova EV, Prokofyev AO, Posikera IN, Morozov AA (2007). Inverted event-related potentials response to illusory contour in boys with autism.. Neuroreport.

